# Mathematical analysis of a lymphatic filariasis model with quarantine and treatment

**DOI:** 10.1186/s12889-017-4160-8

**Published:** 2017-03-16

**Authors:** Peter M. Mwamtobe, Simphiwe M. Simelane, Shirley Abelman, Jean M. Tchuenche

**Affiliations:** 1 School of Computer Science and Applied Mathematics, University of the Witwatersrand, Johannesburg, Private Bag 3, Wits, Johannesburg, 2050 South Africa; 2 DST-NRF Centre of Excellence in Mathematical and Statistical Sciences (CoE-MaSS), University of the Witwatersrand, Johannesburg, Private Bag 3, WitsJohannesburg, 2050 South Africa; 3 Department of Mathematics and Statistics, University of Malawi, Chichiri, Blantyre, Malawi

**Keywords:** Lymphatic filariasis, Intervention strategies, Latent stage, Reproduction number

## Abstract

**Background:**

Lymphatic filariasis is a globally neglected tropical parasitic disease which affects individuals of all ages and leads to an altered lymphatic system and abnormal enlargement of body parts.

**Methods:**

A mathematical model of lymphatic filariaris with intervention strategies is developed and analyzed. Control of infections is analyzed within the model through medical treatment of infected-acute individuals and quarantine of infected-chronic individuals.

**Results:**

We derive the effective reproduction number, $\mathcal {R}_{0},$ and its interpretation/investigation suggests that treatment contributes to a reduction in lymphatic filariasis cases faster than quarantine. However, this reduction is greater when the two intervention approaches are applied concurrently.

**Conclusions:**

Numerical simulations are carried out to monitor the dynamics of the filariasis model sub-populations for various parameter values of the associated reproduction threshold. Lastly, sensitivity analysis on key parameters that drive the disease dynamics is performed in order to identify their relative importance on the disease transmission.

## Background

**Fig. 1:**
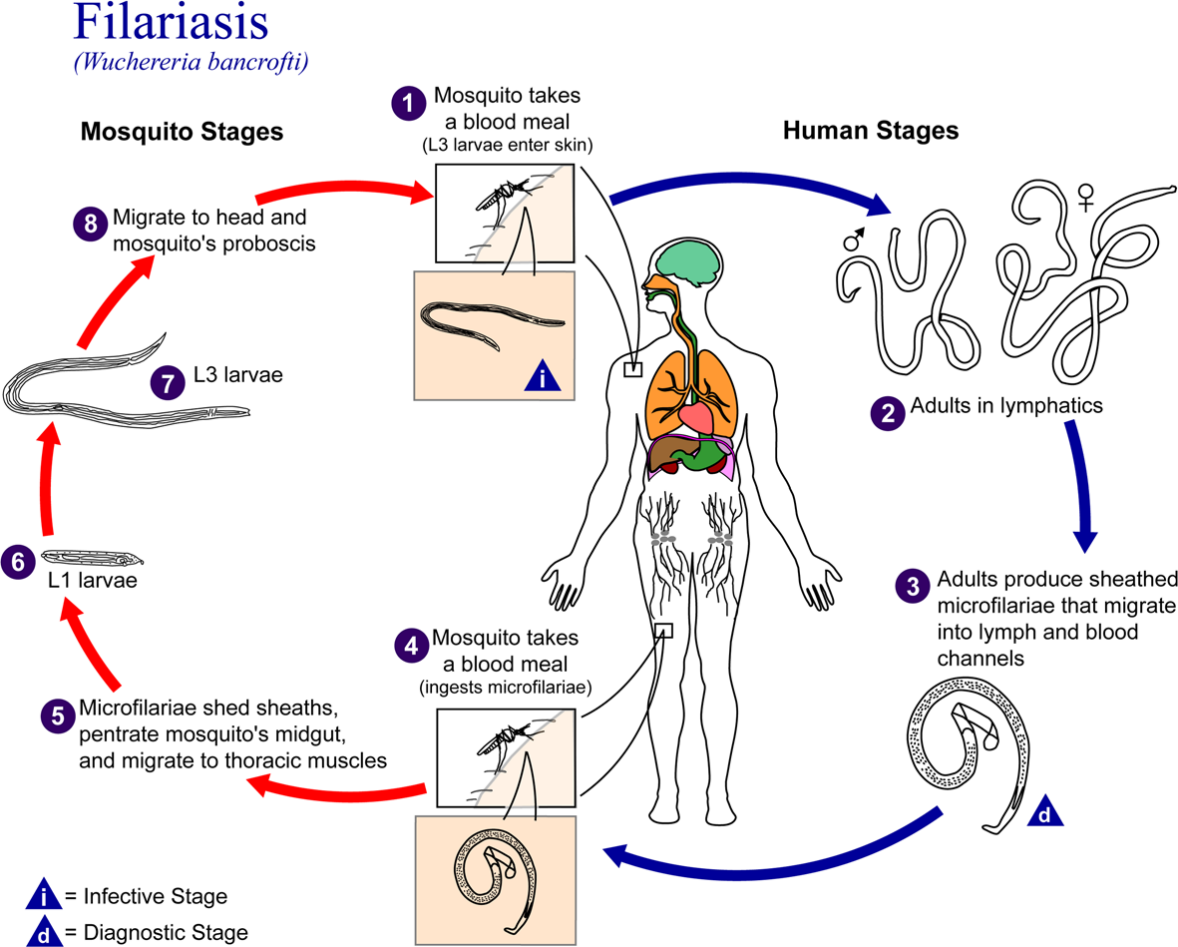
Nematodes (roundworms.) Life cycle of roundworms which cause lymphatic filariasis

Lymphatic Filariasis commonly known as elephantiasis is a globally neglected tropical parasitic disease caused by a thread-like worms of the *Filarioidea* type (*Wuchereria bancrofti, Brugia malayi* and *Brugia timori*) [1]. The most common of these, *Wuchereria bancrofti*, is a round worm that mainly infects the lymphatic system. Lymphatic filariasis involves asymptomatic, acute and chronic conditions with the majority of infections being asymptomatic [2]. Nearly 1.4 billion people in 73 countries worldwide are threatened by the disease of which over 120 million individuals are currently infected [3]. The round worm (nematode) is spread from person to person via a mosquito vector and infected individuals can suffer from chronic conditions such as lymphedema, elephantiasis and, in men, swelling of the scrotum called hydrocele [1, 2]. A description of the microfilariae life cycle is depicted in Fig. 1.

Lymphatic filariasis is still a major public health problem in Africa, South America and Asia despite existing knowledge of the disease pathology and global treatment campaign [3] with drugs such as Diethylcarbamazine plus Albendazole and Ivermectin plus Albendazole that kill the microfilariae and some of the adult worms. There is not enough evidence on effectiveness of the drug Albendazole, alone or in combination, for killing or interrupting transmission of threadlike worms that cause lymphatic filariasis [4]. Some studies have shown that treatment with Doxycycline could completely kill microfilariae [2].

Eradication of this disease has been a great challenge [3, 5]. Thus, investigating the impact of combined intervention strategies of treatment and quarantine of chronically infected persons is viable. Chronically infected individuals may not transmit infection when quarantined from the rest of the population [6, 7]. Insecticide treated-bed nets (ITNs) and sleeping in indoor residual sprayed houses (IRS) could reduce contact between humans (especially microfilariae carriers) and mosquito vectors [8]. Treatment still remains the first line of defense to combat the disease, despite uncertainty about the microfilarial prevalence threshold level below which transmission cannot be sustained even in the absence of any treatments [3, 9].

Compartmental mathematical models of lymphatic filariasis abound in the literature [6, 9–11]. Two general simulation models of lymphatic filariasis transmission and control used to support decision-making are - the population-based deterministic model (EPIFIL) [12, 13] and - the individual-based stochastic model (LYMFASIM) [14]. However these models have some limitations as they do not account for intervention measures such as quarantine. While EPIFIL uses a constant force-of-infection and accounts for the impact of age structure of the human community [13], LYMFASIM accounts for the role of the immune system in regulating parasite numbers [15]. Luz et al., [16] noted that mathematical modeling of transmission dynamics and cost-effectiveness of neglected diseases can help to maximize the utility of the limited available resources. Bhunu and Mushayabasa [10] considered treatment as the only intervention strategy in their model, while Ottesen et al. [8] presented strategies and tools to control transmission and morbidity of lymphatic filariasis. Although various transmission and control mathematical models of lymphatic filariasis abound in the literature, our proposed model is seemingly new as it includes latent stage, treatment and quarantine of chronically infected persons [8, 17]. The latent stage is included in the model because of different developmental stages the worm undergoes in human and mosquito populations.

The proposed compartmental model is not exhaustive, and here are some limitations: no density dependent and species-specific parasite prevalence [18], additional mortality experienced by infected mosquitoes as a result of carrying filarial infection [19]. Pichon [20] noted that mosquito density-dependent mortality may be associated with increased infection intensity within the mosquito and mass drug administration may lead to an increase in survival of the mosquito population and hence to an increase in transmission in the long-term [20].

In the following sections, we formulate and analyse a deterministic model with two key control measures: quarantine and treatment. Key parameters that influence transmission are identified via sensitivity analysis of the model. Finally, some parameter values are assumed within realistic ranges to support the analytical results, but with one caveat that the model outcomes are not compared with real data.

## Methods: model formulation and description

Human and mosquito populations are divided based on their lymphatic filariasis status. Human sub-populations are susceptible humans *S*_*h*_(*t*), latent stage (not showing signs of lymphatic filariasis) *E*_*h*_(*t*), infected-acute stage *I*_*ha*_(*t*) and infected-chronic stage *I*_*hc*_(*t*), with the total human population given by

1 $$\begin{array}{@{}rcl@{}}  N_{h}(t) = S_{h}(t) + E_{h}(t) + I_{ha}(t) + I_{hc}(t). \end{array} $$

The model is formulated with the assumption that no infection exists at the initial stage, and there is no vertical transmission in both human and mosquito populations [17, 21]. In addition, the model considers one species of worm and one species of mosquito. We also assume that the transmission to mosquito population is from infected-acute and infected-chronic individuals despite the quarantine of some infected-chronic individuals.

The mosquito population is divided into three subgroups: susceptible *S*_*v*_(*t*), exposed *E*_*v*_(*t*) and infected *I*_*v*_(*t*), with the total mosquito population given by

2 $$\begin{array}{@{}rcl@{}}  N_{v}(t) = S_{v}(t) + E_{v}(t) + I_{v}(t). \end{array} $$

**Fig. 2:**
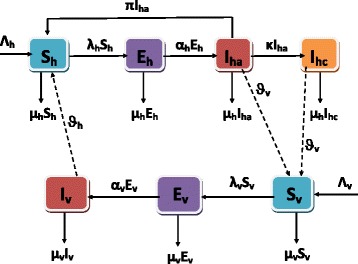
Flowchart for lymphatic filariasis with control strategy and quarantined infected-chronic individuals. The *dash lines* show that the infected mosquitoes (*I*_*v*_) infect the susceptible individuals (*S*_*h*_), the infected-acute individuals (*I*_*ha*_) infect the susceptible mosquitoes (*S*_*v*_) and the infected-chronic individuals (*I*_*hc*_) infect the susecptible mosquitoes (*S*_*v*_)

The recruitment rate of human population is *Λ*_*h*_, while *Λ*_*v*_ is the recruitment rate of the mosquito population. The natural death rates of human and mosquito populations are *μ*_*h*_ and *μ*_*v*_ respectively. These death rates are proportional to the number of each individual or mosquito class. The biting rate of the mosquitoes to humans is *β*. The microfilariae which are found in lymphatic vessels and lymphatic nodes infect susceptible mosquitoes when a mosquito bites infected-acute and infected-chronic individuals at a rate

$$ \lambda_{v}(t) = \displaystyle\frac{\beta\vartheta_{v} (I_{ha}(t) + \theta I_{hc}(t))}{N_{h}(t)}, $$

where *𝜗*_*v*_ is the success rate of microfilariae transmission from human to susceptible mosquitoes and *θ*∈(0,1) accounts for the reduced number of adult microfilariae in humans due to treatment and quarantine of the infected-chronic individuals. The vector will ingest microfilarial differently when it bites humans in the *I*_*ha*_ and *I*_*hc*_ stages. The rate of release *𝜗*_*v*_ *θ* of microfilarial by *I*_*hc*_(*t*) into the vector is different from the rate of release of microfilarial by *I*_*ha*_(*t*). Therefore the microfilariae ingested by vectors during a blood meal depend on the density of microfilariae in humans [11]. Thereafter, susceptible mosquitoes enter the exposed class *E*_*v*_(*t*). During this stage, the microfilariae develop into infective filariform larvae to become infectious, and hence these mosquitoes move into the infected class *I*_*v*_(*t*) at rate *α*_*v*_. The larvae infect the susceptible human host during a subsequent blood meal by the infected mosquitoes at a rate

$$ \lambda_{h}(t) = \displaystyle\frac{\beta \vartheta_{h} I_{v}(t)}{N_{h}(t)}, $$

where *𝜗*_*h*_ is the success rate of transmission of infective filariform larvae from infected mosquitoes *I*_*v*_(*t*) biting susceptible individuals during a blood meal. Individuals during the exposed stage have infective filariform larvae which migrate to lymphatic vessels and lymphatic nodes, and develop into adult worms. The latent individuals progress to infected-acute individuals at a rate *α*_*h*_ when the microfilariae develop into adults which remain in the lymphatic vessels and lymphatic nodes. Furthermore, the infected-acute individuals who progress to chronic condition are quarantined at symptomatic rate *κ* to join the infected-chronic class. Individuals in the infected-acute class, *I*_*ha*_, are screened by health personnel at the rate *n* and treated at the rate *φ*. Treated infected-acute individuals join the susceptible class due to temporary immunity at a rate *π*=*φ* *n*. Figure 2 provides a graphical interpretation of the lymphatic compartmental model (3).

Based on our model description, assumptions, definitions of the state variables and parameters in Table 1, the proposed SEIS lymphatic filariasis model satisfies the following system of nonlinear ordinary differential equations:

3 $$ \left. \begin{array}{llll}  \displaystyle\frac{{dS}_{h}}{dt} &=& \Lambda_{h} + \varphi n I_{ha} - \displaystyle\frac{\beta \vartheta_{h} I_{v} S_{h}}{N_{h}} - \mu_{h} S_{h}\\[0.3cm] \displaystyle\frac{{dE}_{h}}{dt} &=& \displaystyle\frac{\beta \vartheta_{h} I_{v} S_{h}}{N_{h}} - (\alpha_{h} + \mu_{h})E_{h}\\[0.3cm] \displaystyle\frac{{dI}_{ha}}{dt} &=& \alpha_{h} E_{h} - \varphi n I_{ha} - (\kappa + \mu_{h})I_{ha}\\[0.3cm] \displaystyle\frac{{dI}_{hc}}{dt} &=& \kappa I_{ha} - \mu_{h} I_{hc}\\[0.3cm] \displaystyle\frac{{dS}_{v}}{dt} &=& \Lambda_{v} - \displaystyle\frac{\beta\vartheta_{v}(I_{ha} + \theta I_{hc})S_{v}}{N_{h}} - \mu_{v} S_{v}\\[0.3cm] \displaystyle\frac{{dE}_{v}}{dt} &=& \displaystyle\frac{\beta\vartheta_{v} (I_{ha} + \theta I_{hc})S_{v}}{N_{h}} - (\alpha_{v} + \mu_{v})E_{v}\\[0.3cm] \displaystyle\frac{{dI}_{v}}{dt} &=& \alpha_{v} E_{v} - \mu_{v} I_{v} \end{array} \right\}  $$

**Table 1:** The parameters and description for lymphatic filariasis model

Parameter	Description
*Λ*_*h*_	Recruitment rate of human population
*Λ*_*v*_	Recruitment rate of mosquito population
*μ*_*h*_	Natural death rate of human population
*μ*_*v*_	Natural death rate of mosquito population
*β*	Biting rate of the mosquito to humans
*𝜗*_*h*_	Success rate of transmission of infective filariform larvae from mosquitoes to humans
*𝜗*_*v*_	Success rate of microfilariae transmission from infected-acute and infected-chronic
	humans to susceptible mosquitoes
*θ*	Accounts for reduced number of adult microfilariae in humans due to treatment and
	quarantine of the infected chronic individuals
*α*_*h*_	Progression rate of latent individuals to infected-acute individuals
*α*_*v*_	Progression rate from exposed mosquitoes to infected mosquitoes
*κ*	Symptomatic rate of infected-acute individuals progressed to infected-chronic individuals
*n*	Number of screened infected-acute individuals by health personnel
*φ*	Treatment/prevention chemotherapy rate of the infected-acute individuals

## Results

### Invariant region

Both the model state variables and parameters are assumed non-negative for all time *t*≥0. Let ${(S_{h}, E_{h}, I_{ha}, I_{hc}, S_{v}, E_{v}, I_{v})} \in {\mathbb {R}^{7}}$ be any solution of the system with non-negative initial conditions. Applying Birkhoff and Rota’s Theorem [22] on differential inequality, from Eq. (1), we have $N_{h}^{\prime }(t) < \Lambda _{h} - \mu _{h} N_{h}(t)$ as *t*→*∞*, and thus, $0 \leq N_{h}(t) \leq \displaystyle \frac {\Lambda _{h}}{\mu _{h}}.$ Hence the feasible solutions on the human population enter the region

4 $$\begin{array}{@{}rcl@{}}  \Psi_{h} = \left\{(S_{h}, E_{h}, I_{ha}, I_{hc}) \in \mathbb{R}^{4}_{\geq 0} : N_{h}(t) \leq \frac{\Lambda_{h}}{\mu_{h}}\right\}. \end{array} $$

Similarly, it can be shown that the feasible solutions on the mosquito population given by Eq. (2) enter the region

5 $$\begin{array}{@{}rcl@{}}  \Psi_{v} = \left\{(S_{v}, E_{v}, I_{v}) \in \mathbb{R}^{3}_{\geq 0}: N_{v}(t) \leq \frac{\Lambda_{v}}{\mu_{v}} \right\}. \end{array} $$

Therefore, from (4) and (5), the possible solutions of model (3) will enter the the positively invariant region *Ψ*=*Ψ*_*h*_×*Ψ*_*v*_.

### Positivity of the state variables

Since *Ψ* is a positively invariant set under the flow induced by model (3), we now show that every solution with initial condition in $\mathbb {R}^{7}$ remains in that region for *t*>0.

#### **Theorem 1**

The solution set {*S*_*h*_,*E*_*h*_,*I*_*ha*_,*I*_*hc*_,*S*_*v*_,*E*_*v*_,*I*_*v*_}(*t*) of the lymphatic filariasis model (3) with the initial condition {*S*_*h*_,*E*_*h*_,*I*_*ha*_,*I*_*hc*_,*S*_*v*_,*E*_*v*_,*I*_*v*_}(0) is positive for all *t*>0.

#### *Proof*

Let $\tilde {t} = {\text {sup}} \left \{t > 0 : S_{h} > 0, E_{h} > 0, I_{ha} > 0,\right. \ \left.I_{hc} > 0, S_{v} > 0, E > 0, I_{v} > 0 \right \} \in \left [0, t \right ],$ gives $\tilde {t} > 0.$ The first equation of model (3) gives

$$\begin{array}{@{}rcl@{}} \frac{{dS}_{h}}{dt} = \Lambda_{h} + \varphi n I_{ha} - \frac{\beta \vartheta_{h} I_{v} S_{h}}{N_{h}} - \mu_{h} S_{h} \geq -(\lambda_{h} + \mu_{h})S_{h}. \end{array} $$

Intergrating with respect to *t* gives

$$\begin{array}{@{}rcl@{}} \frac{d}{dt} \left[S_{h}(t)e^{\displaystyle\int_{0}^{t} \lambda_{h}(s)ds + \mu_{h} t} \right] \geq e^{\displaystyle\int_{0}^{t} \lambda_{h}(s)ds + \mu_{h} t}. \end{array} $$

Therefore,

$$\begin{aligned} &S_{h}(\overline{t})e^{\displaystyle\int_{0}^{\overline{t}}\left\lbrace \lambda_{h}(s)ds\right\rbrace + \mu_{h} \overline{t}} - S_{h}(0) \geq\\ &\displaystyle\int_{0}^{\overline{t}} e^{\displaystyle\int_{0}^{t^{*}}{\lambda_{h}(w)dw} + u_{h} t^{*}}dt^{*}, \end{aligned} $$

so that

$$\begin{array}{@{}rcl@{}} S_{h}(\overline{t}) &\geq& S_{h}(0) e^{-\left(\displaystyle\int_{0}^{\overline{t}}\lambda_{h}(s)ds + \mu_{h}\overline{t} \right)} ~ \\[0.2cm] &&+e^{-\left(\displaystyle\int_{0}^{\overline{t}}\lambda_{h}(s)ds + \mu_{h}\overline{t} \right)}\\ &&\times\left\{ \displaystyle\int_{0}^{\overline{t}}{ e^{\displaystyle\int_{0}^{t^{*}}\lambda_{h}(w)dw + \mu_{h}t^{*}} dt^{*}} \right\} > 0. \end{array} $$

Hence *S*_*h*_ is always positive for *t*>0.The second equation of model (3) gives

$$\begin{array}{@{}rcl@{}} \frac{{dE}_{h}}{dt} &=& \frac{\beta \vartheta_{h} I_{v} S_{h}}{N_{h}} - (\alpha_{h} + \mu_{h})E_{h}\\[0.3cm] \displaystyle\int \frac{1}{E_{h}}{dE}_{h} &\geq & - \displaystyle\int (\alpha_{h} + \mu_{h})dt\\[0.3cm] \Rightarrow E_{h}(t) &\geq & E_{h}(0) e^{-(\alpha_{h} + \mu_{h})t} > 0. \end{array} $$

Similarly it can be shown that *I*_*ha*_>0,*I*_*hc*_>0,*S*_*v*_>0,*E*_*v*_>0,*I*_*v*_>0 for *t*>0. □

### Existence and stability of steady-state solutions

The disease-free equilibrium (DFE) of the lymphatic filariasis model (3) denoted by **E**_0_ is given by

$$\begin{array}{@{}rcl@{}} \mathbf{E}_{0} = \left(S_{h}^{*}, E_{h}^{*}, I_{ha}^{*}, I_{hc}^{*}, S_{v}^{*}, E_{v}^{*}, I_{v}^{*} \right) = \left(\frac{\Lambda_{h}}{\mu_{h}}, 0, 0, 0, \frac{\Lambda_{v}}{\mu_{v}}, 0, 0 \right). \end{array} $$

The effective reproduction number is obtained by using the next generation matrix [23]. Let

$$\mathcal{F} = \left[\begin{array}{c} \frac{\beta \vartheta_{h} I_{v} S_{h}}{N_{h}} \\[0.3cm] 0 \\[0.3cm] 0 \\[0.3cm] \frac{\beta \vartheta_{v} (I_{ha} + \theta I_{hc})S_{v}}{N_{h}} \\[0.3cm] 0 \end{array}\right] $$

and

$$\mathrm{V} = \left[\begin{array}{ccccc} \alpha_{h} + \mu_{h} & 0 & 0 & 0 & 0 \\[0.3cm] -\alpha_{h} & \varphi n + \kappa + \mu_{h} & 0 & 0 & 0 \\[0.3cm] 0 & -\kappa & \mu_{h} & 0 & 0 \\[0.3cm] 0 & 0 & 0 & \alpha_{v} + \mu_{v} & 0 \\[0.3cm] 0 & 0 & 0 & -\alpha_{v} & \mu_{v} \end{array}\right]. $$

The effective reproduction number is the spectral radius *ρ*(FV^−1^) and the resulting expression is given by

6 $$ {\begin{aligned} \mathcal{R}_{0} = \frac{\beta \alpha_{h} \vartheta_{h} \alpha_{v} \Lambda_{v} \vartheta_{v} \left(\theta \kappa +\mu_{h}\right)}{\mu_{v} \sqrt{\alpha_{h} \Lambda_{h} \vartheta_{h} \alpha_{v} \Lambda_{v} \vartheta_{v} \left(\alpha_{h}+\mu_{h}\right) \left(\alpha_{v}+\mu_{v}\right) \left(\theta \kappa +\mu_{h}\right) \left(\mu_{h}+\kappa +n \varphi \right)}}, \end{aligned}}  $$

which is the number of secondary lymphatic filariasis infections caused by one infectious individual/mosquito during the infectious period in a completely susceptible population. The effective reproduction number is not only important for describing how fast the disease could spread, but can also provide information for controlling and preventing the spread of the disease [24].

#### Local stability of the disease-free equilibrium

Local stability of the DFE can be established from Theorem 2 in [23].

##### **Lemma 2**

The DFE for the lymphatic filariasis model (3) is locally asymptotically stable if $\mathcal {R}_{0} < 1$ and unstable when $\mathcal {R}_{0} > 1.$

#### Global stability of the disease-free equilibrium

The system of Eq. (3) is broken into subsystems such that *X*_1_=(*S*_*h*_,*S*_*v*_) which denotes the number of susceptible individuals and susceptible mosquitoes, and *Y*_1_=(*E*_*h*_,*I*_*ha*_,*I*_*hc*_,*E*_*v*_,*I*_*v*_) which denotes the number of exposed and infected individuals and mosquitoes. Hence the model system (3) now reduces to

$$\left. \begin{array}{llllll} \frac{{dX}_{1}}{dt} &=& \mathbf{F}(X_{1}, Y_{1})\\[0.3cm] \frac{{dY}_{1}}{dt} &=& \mathbf{G}(X_{1}, Y_{1}) \end{array} \right\} \text{~where~} X_{1} \in \mathbb{R}^{2}_{+},~ Y_{1} \in \mathbb{R}_{+}^{5}. $$

This could further be simplified by identifying *X*_1_ with (*X*_1_,0) and *Y*_1_ with (0,*Y*_1_) in $\mathbb {R}_{+}^{2} \times \mathbb {R}_{+}^{5}.$ Hence we obtain the reduced system $\frac {{dX}_{1}}{dt} = \mathbf {F}(X_{1}, 0)$ as

7 $$ \left. \begin{array}{lllll}  \frac{{dS}_{h}}{dt} &=& \Lambda_{h} - \mu_{h} S_{h}\\[0.3cm] \frac{{dS}_{v}}{dt} &=& \Lambda_{v} - \mu_{v} S_{v} \end{array} \right\}.  $$

Therefore $X^{*}_{1} = (S_{h}^{*}, S_{v}^{*}) = \left (\frac {\Lambda _{h}}{\mu _{h}}, \frac {\Lambda _{v}}{\mu _{h}}\right)$ is a global asymptotically stable equilibrium for the reduced system $\frac {{dX}_{1}}{dt} = \mathbf {F}(X_{1}, 0).$ This is verified by integrating the first equation of the reduced system (7) with respect to *t*. We obtain $S_{h}(t) = \frac {\Lambda _{h}}{\mu _{h}} + \left (S_{h}(0) - \frac {\Lambda _{h}}{\mu _{h}}\right)e^{-\mu _{h} t}$ which approaches $\frac {\Lambda _{h}}{\mu _{h}} \text {~as~} t \rightarrow \infty.$ Similarly integrating the second equation of the reduced system (7) with respect to *t*, gives $S_{v}(t) = \frac {\Lambda _{v}}{\mu _{v}} + \left (S_{v}(0) - \frac {\Lambda _{v}}{\mu _{v}}\right)e^{-\mu _{v} t}$ which approaches $\frac {\Lambda _{v}}{\mu _{v}} \text {~as~} t \rightarrow \infty.$

Further **G**(*X*_1_,*Y*_1_) satisfies the two conditions given as assumptions **H3** and **H4** in [25] namely: **G**(*X*_1_,0)=0 and $\mathbf {G}(X_{1}, Y_{1}) = A^{*} Y_{1} - \tilde {\mathbf {G}}(X_{1}, Y_{1}), \text {~where~} \tilde {\mathbf {G}}(X_{1}, Y_{1}) \geq 0 \in \Psi $ such that

$${\begin{aligned} A^{*} &= D_{Y} \mathbf{G}(X_{1}, 0)\\ &= \left[\begin{array}{ccccc} -(\alpha_{h} + \mu_{h}) & 0 & 0 & 0 & \beta \vartheta_{h} \\[0.2cm] \alpha_{h} & -(\varphi n + \kappa + \mu_{h}) & 0 & 0 & 0 \\[0.2cm] 0 & \kappa & -\mu_{h} & 0 & 0 \\[0.2cm] 0 & \frac{\beta \vartheta_{v} \Lambda_{v} \mu_{h}}{\Lambda_{h} \mu_{v}} & \frac{\beta \vartheta_{v} \theta \Lambda_{v} \mu_{h}}{\Lambda_{h} \mu_{v}} & -(\alpha_{v} + \mu_{v}) & 0 \\[0.2cm] 0 & 0 & 0 & \alpha_{v} & -\mu_{v} \end{array}\right] \end{aligned}} $$

and

$$\tilde{\mathbf{G}}(X_{1}, Y_{1}) = \left[\begin{array}{c} \beta \vartheta_{h} I_{v} \left(1 - \frac{S_{h}}{N_{h}}\right)\\[0.3cm] 0 \\[0.3cm] 0 \\[0.3cm] \beta \vartheta_{v} \left(I_{ha} + \theta I_{hc} \right)\left(\frac{\Lambda_{v} \mu_{h}}{\mu_{v} \Lambda_{h}} - \frac{S_{v}}{N_{h}} \right) \\[0.3cm] 0 \end{array}\right]. $$

Here we assume a steady-state value of the total human population $N_{h} = \frac {\Lambda _{h}}{\mu _{h}}$ and total mosquito population $N_{v} = \frac {\Lambda _{v}}{\mu _{v}}.$ Therefore the term $\beta \vartheta _{v} \left (I_{ha} + \theta I_{hc} \right)\left [\frac {\Lambda _{v} \mu _{h}}{\mu _{v} \Lambda _{h}} - \frac {S_{v}}{N_{h}} \right ] \in \tilde {\mathbf {G}}(X_{1}, Y_{1})$ is non-negative. Thus the DFE is globally asymptotically stable since $\tilde {\mathbf {G}}(X_{1}, Y_{1})$ is non-negative. The global stability of **E**_0_ excludes any possibility of the phenomenon of backward bifurcation, that is the co-existence of a stable disease-free equilibrium with a stable endemic equilibrium [26].

#### Existence of endemic equilibria

To determine the existence of an equilibrium for which filariasis is endemic in the population defined by $\mathcal {E}_{0}^{*} = (S_{h}^{*}, E_{h}^{*}, I_{ha}^{*}, I_{hc}^{*}, S_{v}^{*}, E_{v}^{*}, I_{v}^{*})$, the system (3) is solved in terms of the force of infection at steady-state ($\lambda _{h}^{*}$), given by

8 $$ \lambda_{h}^{*}(t) = \frac{\beta \vartheta_{h} I_{v(t)}^{*}}{N_{h(t)}^{*}}.  $$

Solving the system at an arbitrary equilibrium, we have

9 $$ \left. \begin{array}{llll}  0 &=& \Lambda_{h} + \varphi n I_{ha}^{*} - \lambda_{v}^{*} S_{h}^{*} - \mu_{h} S_{h}^{*}\\ 0 &=& \lambda_{v}^{*} S_{h}^{*} - (\alpha_{h} + \mu_{h})E_{h}^{*}\\ 0 &=& \alpha_{h} E_{h}^{*} - \varphi n I_{ha}^{*} - (\kappa + \mu_{h})I_{ha}^{*}\\ 0 &=& \kappa I_{ha}^{*} - \mu_{h} I_{hc}^{*}\\ 0 &=& \Lambda_{v} - \lambda_{h}^{*} S_{v}^{*} - \mu_{v} S_{v}^{*}\\ 0 &=& \lambda_{h}^{*} S_{v}^{*} - (\alpha_{v} + \mu_{v})E_{v}^{*}\\ 0 &=& \alpha_{v} E_{v}^{*} - \mu_{v} I_{v}^{*} \end{array} \right\}.  $$

Thus

$$ {\begin{aligned} S_{h}^{*} &= \frac{\Lambda_{h} \left(\alpha_{h}+\mu_{h}\right) \left(\mu_{h}+\kappa +n \varphi \right)}{\left(\alpha_{h}+\mu_{h}\right) \left(\lambda^{*}_{h}+\mu_{h}\right) \left(\mu_{h}+\kappa +n \varphi \right)-n \varphi \alpha_{h} \lambda_{h}^{*}},\\ E_{h}^{*} &= \frac{\lambda_{h}^{*} \Lambda_{h} \left(\mu_{h}+\kappa +n \varphi \right)}{\alpha_{h} \left(\lambda_{h}^{*} \left(\mu_{h}+\kappa \right)+\mu_{h} \left(\mu_{h}+\kappa +n \varphi \right)\right)+\mu_{h} \left(\lambda_{h}^{*} +\mu_{h}\right) \left(\mu_{h}+\kappa +n \varphi \right)},\\ I_{ha}^{*} &= \frac{\alpha_{h} \lambda_{h}^{*} \Lambda_{h}}{\alpha_{h} \left(\lambda_{h}^{*} \left(\mu_{h}+\kappa \right)+\mu_{h} \left(\mu_{h}+\kappa +n \varphi \right)\right)+\mu_{h} \left(\lambda_{h}^{*} +\mu_{h}\right) \left(\mu_{h}+\kappa +n \varphi \right)},\\ I_{hc}^{*} &= \frac{\kappa \alpha_{h} \lambda_{h}^{*} \Lambda_{h}}{\mu_{h} \left(\alpha_{h} \left(\lambda_{h}^{*} \left(\mu_{h}+\kappa \right)+\mu_{h} \left(\mu_{h}+\kappa +n \varphi \right)\right)+\mu_{h} \left(\lambda_{h}^{*} +\mu_{h}\right) \left(\mu_{h}+\kappa +n \varphi \right)\right)},\\ S_{v}^{*} &= \frac{\Lambda_{v}}{\lambda_{v}^{*} +\mu_{v}},\\ E_{v}^{*} &= \frac{\lambda_{v}^{*} \Lambda_{v} }{\left(\alpha_{v} +\mu_{v}\right) \left(\lambda_{v}^{*} +\mu_{v}\right)},\\ I_{v}^{*} &= \frac{\alpha_{v} \lambda_{v}^{*} \Lambda_{v}}{\mu_{v} \left(\alpha_{v}+\mu_{v}\right) \left(\lambda_{v}^{*} +\mu_{v}\right)}, \end{aligned}} $$

and $\lambda _{h}^{*} = \beta \vartheta _{h} \frac {I_{v}^{*}}{S_{h}^{*} + E_{h}^{*} + I_{ha}^{*} + I_{hc}^{*}}$. Substituting $I_{v}^{*}$ and the above solutions into the expression for $\lambda _{h}^{*}$, we have

$$ \lambda_{h}^{*} = \frac{\beta \mu_{h} \vartheta_{h} \alpha_{v} \lambda_{v}^{*} \Lambda_{v}}{\Lambda_{h} \mu_{v} \left(\alpha_{v}+\mu_{v}\right) \left(\lambda_{v}^{*} +\mu_{v}\right)}.  $$

This can be written as

10 $$ \lambda_{h}^{*} (A \lambda_{h}^{*} + B) = 0,  $$

where

$$ {\begin{aligned} A &= \Lambda_{h} \mu_{v} \left(\alpha_{v}+\mu_{v}\right) \left(\mu_{h} \mu_{v} \left(\mu_{h}+\kappa +n \varphi \right)\right.\\ &\left.\quad+\alpha_{h} \left(\beta \vartheta_{v} \left(\theta \kappa +\mu_{h}\right)+\mu_{v} \left(\mu_{h}+\kappa \right)\right)\right), \\ B &= \mu_{h} \Lambda_{h} (\mu_{h}+\kappa +n \varphi)(\alpha_{h}+\mu_{h}) (\alpha_{v}+\mu_{v})\mu_{v}^{2} (\mathcal{R}_{0}^{2} - 1). \end{aligned}} $$

The root $\lambda _{h}^{*} = 0$ corresponds to the DFE and its stability has already been established in Lemma 2. It is clear that *A*>0, and *B*>0 if and only if $\mathcal {R}_{0} > 1$. Thus the linear system would have a unique positive solution given by $\lambda _{h}^{*} = B /A$. The components of the endemic equilibrium, $\mathcal {E}_{0}^{*}$, are then determined by substituting $\lambda _{h}^{*} = B / A$. For $\mathcal {R}_{0} < 1$, *B*<1. Thus, the force of infection ($\lambda _{h}^{*}$) at steady-state is negative (which is biologically meaningless). Hence, the model system has no positive equilibria in this case i.e when $\lambda _{h}^{*} < 0$. This result is summarized in the following Lemma.

##### **Lemma 3**

The lymphatic filariasis model has a unique endemic equilibrium whenever $\mathcal {R}_{0} > 1$, and no endemic equilibrium otherwise.

### Sensitivity analysis and model simulations

The following local sensitivity analysis is closely related to that in [27]. Expressions for the sensitivity indices of the endemic equilibrium are complex, and since our focus is on disease transmission and not prevalence, we neither derive expressions nor numerically calculate sensitivity indices of the endemic equilibrium.

#### Sensitive indices of ${\mathcal {R}_{0}}$

The sensitivity indices allow us to measure the relative change in a state variable when a parameter changes [27]. The normalized forward sensitivity index [27] of a variable, *ψ*, that depends differentiably on a parameter, *p*, is defined as:

11 $$ \Upsilon_{p}^{\psi} = \frac{\partial \psi}{\partial p}. \frac{p}{\psi}.  $$

Next, we evaluate the sensitivity indices at the parameter values given in Table 2. The resulting sensitivity indices are shown in Table 3.

**Table 2:** Values and ranges for parameters for the lymphatic filariasis model (3)

Parameter	Value	Source	Parameter	Value	Source
*μ*_*h*_	0.000039	[28, 29]	*Λ*_*h*_	2500	[6, 30]
*μ*_*v*_	0.1429	[29, 30]	*Λ*_*v*_	1000	[30]
*κ*	10	Assumed	*β*	250	[6]
*φ*	0.7	Assumed	*n*	200	[6]
*𝜗*_*h*_	0.01	[6]	*𝜗*_*v*_	0.1	[6]
*α*_*h*_	0.0238	Assumed	*α*_*v*_	0.0555	Assumed
*θ*	0.0555	[30]

**Table 3:** Sensitivity indices of $\mathcal {R}_{0}$ to parameters for the lymphatic filariasis model, evaluated at the parameter values given in Table 2

$\mathcal {R}_{0}$		
Parameter	$\Upsilon _{p}^{\psi }$	Sensitivity index
*Λ*_*h*_	$-\frac {1}{2}$	-0.5
*μ*_*v*_	$\frac {1}{2} \left (\frac {\alpha _{v}}{\alpha _{v}+\mu _{v}}-3\right)$	-1.36013
*Λ*_*v*_	$\frac {1}{2}$	0.5
*κ*	$\frac {1}{2} \kappa \left (\frac {\theta }{\theta \kappa +\mu _{h}}-\frac {1}{\mu _{h}+\kappa +n \varphi }\right)$	0.462806
*β*	1	1
*φ*	$-\frac {n \varphi }{2 \left (\mu _{h}+\kappa +n \varphi \right)}$	-0.466545
*n*	$-\frac {n \varphi }{2 \left (\mu _{h}+\kappa +n \varphi \right)}$	-0.466545
*𝜗*_*h*_	$\frac {1}{2}$	0.5
*𝜗*_*v*_	$\frac {1}{2}$	0.5
*α*_*h*_	$\frac {\mu _{h}}{2 \left (\alpha _{h}+\mu _{h}\right)}$	0.0000819193
*α*_*v*_	$\frac {\mu _{v}}{2 \left (\alpha _{v}+\mu _{v}\right)}$	0.360131
*θ*	$\frac {\theta \kappa }{2 \theta \kappa +2 \mu _{h}}$	0.49613

The parameters are ordered from most to least sensitive for each $\mathcal {R}_{0}$

The most sensitive parameter to $\mathcal {R}_{0}$ is the mosquitoes natural death rate, *μ*_*v*_ ($\Upsilon _{\mu _{v}}^{\mathcal {R}_{0}} = -1.360$). This is followed by the mosquito per capita biting rate, *β* ($\Upsilon _{\beta }^{\mathcal {R}_{0}} = 1$). Reducing this parameter would have a huge effect on filarisis transmission regardless of other parameter values. We have that $\Upsilon _{\beta }^{\mathcal {R}_{0}} = 1$, then decreasing (or increasing) *β* by 10*%* decreases (or increases) $\mathcal {R}_{0}$ by 10*%*.

Other key parameters include the success rate of microfilariae transmission from humans to susceptible mosquitoes, *𝜗*_*v*_, as well as the success rate of transmission of infective larvae from infected biting mosquitoes to susceptible individuals during a blood meal, *𝜗*_*h*_. The sensitivity indices of these two parameters are equal and independent of other system parameters. With $\Upsilon _{\alpha _{v}}^{\mathcal {R}_{0}} = 8.19 \text {~x~} 10^{-5}$, the progression rate of mosquitoes from exposed to infectious state, *α*_*v*_, is the least sensitive parameter. For ethical reasons, one should only attempt to decrease human death rate and for this reason, we do not calculate the sensitivity index related to individuals natural death.

For the mosquito recruitment rate *Λ*_*v*_, the filariasis reproduction number, $\mathcal {R}_{0}$, increases as *Λ*_*v*_ increases. If the mosquito recruitment increases, so does the mosquito death rate because the environment can only support a certain number of mosquitoes. Further, when the mosquito recruitment rate *Λ*_*v*_ is equal to the death rate *μ*_*v*_, the mosquito population is at equilibrium. If 1/*Λ*_*v*_ is the life span of the mosquitoes, then increasing *Λ*_*v*_ reduces their life span. Reducing the life span of the vector population reduces $\mathcal {R}_{0},$ as more infected mosquitoes die before they become infectious.

#### Analysis of $\boldsymbol {\mathcal {R}_{0}}$

The objective here is to determine, using the threshold quantity $\mathcal {R}_{0}$, whether or not activities provided to quarantined infected-chronic individuals (modelled by *κ*) and treatment (modelled by *φ*) of infected-acute individuals can lead to the elimination of lymphatic filariasis in the community. It is evident from (6) that

12 $$ {\begin{aligned} &\lim \limits_{\kappa \to 1} \mathcal{R}_{0}\\&\quad= \frac{\beta \alpha_{h} \vartheta_{h} \alpha_{v} \Lambda_{v} \vartheta_{v} \left(\mu_{h}+\theta \right)}{\mu_{v} \sqrt{\alpha_{h} \Lambda_{h} \vartheta_{h} \alpha_{v} \Lambda_{v} \vartheta_{v} \left(\alpha_{h}+\mu_{h}\right) \left(\mu_{h}+\theta \right) \left(\alpha_{v}+\mu_{v}\right) \left(\mu_{h}+n \varphi +1\right)}},  \end{aligned}}  $$

13 $$ {\begin{aligned} &\lim \limits_{\varphi \to 1} \mathcal{R}_{0}\\ &\quad= \frac{\beta \alpha_{h} \vartheta_{h} \alpha_{v} \Lambda_{v} \vartheta_{v} \left(\theta \kappa +\mu_{h}\right)}{\mu_{v} \sqrt{\alpha_{h} \Lambda_{h} \vartheta_{h} \alpha_{v} \Lambda_{v} \vartheta_{v} \left(\alpha_{h}+\mu_{h}\right) \left(\alpha_{v}+\mu_{v}\right) \left(\theta \kappa +\mu_{h}\right) \left(\mu_{h}+\kappa +n\right)}}.  \end{aligned}}  $$

**Fig. 3:**
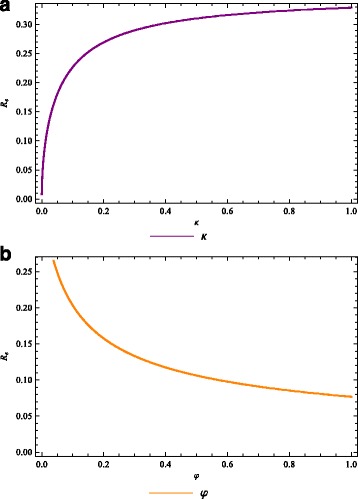
Quarantine and treatment of individuals. The lymphatic filariasis reproduction number $\mathcal {R}_{0}$ as a function of the **a** quarantine (morbidity control) rate *κ*, and **b** function of the treatment rate *φ*

Thus, a sufficient effective quarantine programme (morbidity management and disability prevention activities) that focuses on quarantining infected individuals in the *I*_*ha*_ stage (at a high rate, *κ*→1) can lead to effective disease control if it results in the right hand side of (12) being less than unity. Likewise, for an effective treatment program, the right hand side of (13) should be less than unity. The profiles of $\mathcal {R}_{0}$ as a function of the quarantine rate, *κ*, and treatment rate, *φ*, are depicted in Fig. 3 a and 3 b.

For the set of parameter values used, the strategy that focuses on treating the infected-acute individuals alone can dramatically reduce $\mathcal {R}_{0}$ from around $\mathcal {R}_{0} = 0.264$ to $\mathcal {R}_{0} = 0.077.$ The quarantine strategy increases $\mathcal {R}_{0} = 9.795 \text {~x~} 10^{-3}$ to $\mathcal {R}_{0} = 0.329$. Thus, lymphatic filariasis in the community could be reduced more slowly in the latter case, but will be eliminated faster in the former case.

**Fig. 4:**
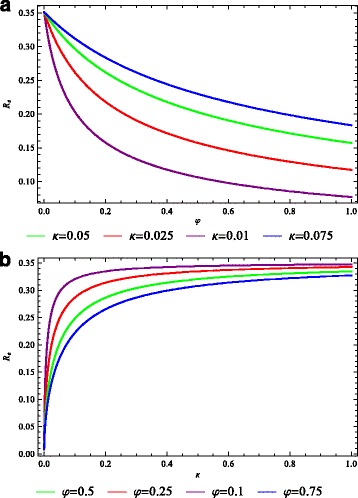
Reproduction number $\mathcal {R}_{0}$. The lymphatic filariasis effective reproduction number as a function of the (**a**) quarantine rate *κ* for different values of treatment rate *φ*, and **b** treatment rate *φ* for different values of quarantine (morbidity control) rate *κ*

Figure 4 a shows that the combined strategy of activities provided to quarantined infected-chronic individuals with an effective treatment of infected-acute individuals reduces $\mathcal {R}_{0}$ to values far below unity than when each strategy is applied singly. Figure 4 b shows that with an effective treatment strategy, increasing the quarantine rate does not necessary reduce the burden of filariasis in the community.

#### $\boldsymbol {\mathcal {R}_{0}}$ as a function ***κ*** and ***φ***

The lymphatic filariasis burden in the community is evaluated by computing the partial derivatives of $\mathcal {R}_{0}$ with respect to the quarantine and treatment parameters (*κ* and *φ* respectively). This gives

14 $$\begin{array}{*{20}l} \frac{\partial \mathcal{R}_{0}}{\partial \kappa} &= \frac{\beta \alpha_{h} \vartheta_{h} \alpha_{v} \Lambda_{v} \vartheta_{v} \left((\theta -1) \mu_{h}+\theta n \varphi \right)}{2 \mu_{v} \left(\mu_{h}+\kappa +n \varphi \right) \sqrt{\alpha_{h} \Lambda_{h} \vartheta_{h} \alpha_{v} \Lambda_{v} \vartheta_{v} \left(\alpha_{h}+\mu_{h}\right) \left(\alpha_{v}+\mu_{v}\right) \left(\theta \kappa +\mu_{h}\right) \left(\mu_{h}+\kappa +n \varphi \right)}},  \end{array} $$

15 $$\begin{array}{*{20}l} \frac{\partial \mathcal{R}_{0}}{\partial \varphi} &= -\frac{\beta n \alpha_{h} \vartheta_{h} \alpha_{v} \Lambda_{v} \vartheta_{v} \left(\theta \kappa +\mu_{h}\right)}{2 \mu_{v} \left(\mu_{h}+\kappa +n \varphi \right) \sqrt{\alpha_{h} \Lambda_{h} \vartheta_{h} \alpha_{v} \Lambda_{v} \vartheta_{v} \left(\alpha_{h}+\mu_{h}\right) \left(\alpha_{v}+\mu_{v}\right) \left(\theta \kappa +\mu_{h}\right) \left(\mu_{h}+\kappa +n \varphi \right)}}.  \end{array} $$

Consider the case when *φ*=0 (there is no treatment but only quarantine). It follows that $\frac {\partial \mathcal {R}_{0}}{\partial \kappa } < 0$ if

16 $$ {\begin{aligned} \frac{\beta \alpha_{h} \vartheta_{h} \alpha_{v} \Lambda_{v} \vartheta_{v} \left((\theta -1) \mu_{h}+\theta n \varphi \right)}{2 \mu_{v} \left(\mu_{h}+\kappa +n \varphi \right) \sqrt{\alpha_{h} \Lambda_{h} \vartheta_{h} \alpha_{v} \Lambda_{v} \vartheta_{v} \left(\alpha_{h}+\mu_{h}\right) \xi}} &< 0 \\[0.15cm] (\theta - 1)\mu_{h} + \theta n \varphi &< 0 \\[0.15cm] \theta < \triangle_{I} &= \frac{\mu_{h}}{\mu_{h}+n \varphi } \end{aligned}}  $$

where *ξ*=(*α*_*v*_+*μ*_*v*_)(*θ* *κ*+*μ*_*h*_)(*μ*_*h*_+*κ*+*n* *φ*).

##### **Lemma 4**

The targeted quarantine strategy of infected-acute individuals will have a positive impact if *θ*<△_*I*_, no impact if *θ*=△_*I*_, and will have detrimental impact in *θ*>△_*I*_.

Similarly, $\frac {\partial \mathcal {R}_{0}}{\partial \varphi } < 0$ if

17 $$ \theta > \triangle_{T} = -\frac{\mu}{\kappa}.  $$

Thus, we have

##### **Lemma 5**

The targeted treatment of infected-acute individuals will have positive impact if *θ*>△_*T*_, no impact if *θ*=△_*T*_ and negative impact if *θ*<△_*T*_.

Note that if conditions (16) and (17) are invalid, then application of the activities provided to quarantined infected-chronic individuals and treatment strategies would increase the burden of filariasis in the community (since it increases $\mathcal {R}_{0}$). Treatment would increase the disease burden if it fails to reduce the infectiousness of those treated below a certain threshold (*θ*>△_*T*_ if treatment of infected-acute individuals is targeted or *θ*<△_*I*_ if quarantine is targeted).

#### Model simulations

**Fig. 5:**
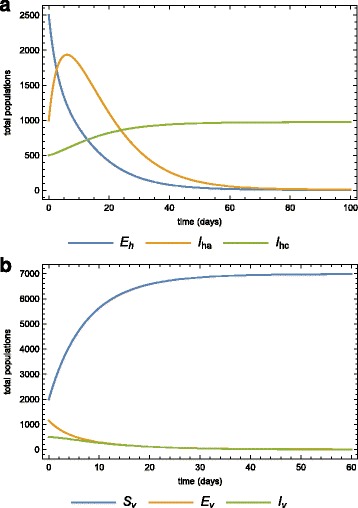
Parameter values simulations. Simulations of model (3) for the parameter values in Table 2 for **a** the human, **b** the vector sub-populations over time

Numerical simulations of the model system (3) are carried out using Wolfram Mathematica 9.0 to illustrate some of the analytical results. Parameter values used for the model simulations are provided in Table 2, some of these were obtained from the literature [27–30] while others were assumed (within realistic range) for the purpose of simulations. The dynamics of the human and mosquito populations when both treatment and quarantine are employed, are depicted in Fig. 5 and 5 b, respectively. The effects of increasing the infected-acute individuals quarantine rate as well as scaling up the treatment of acute-infected individuals on the dynamics of the whole population are explored. Two scenarios are investigated: (1) we assume that a population is invaded by infected mosquitoes, and (2) we assume that a population is invaded by acute-infected humans.

##### Case 1.

**Fig. 6:**
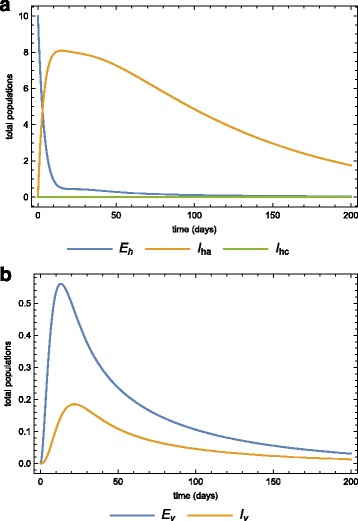
Population without any intervention strategy. The dynamics of the **a** human, and **b** vector sub-populations when there is no medical treatment (prevention chemotherapy) and no quarantine (morbidity management and disability prevention) after the invasion of 10 infected individuals

**Fig. 7:**
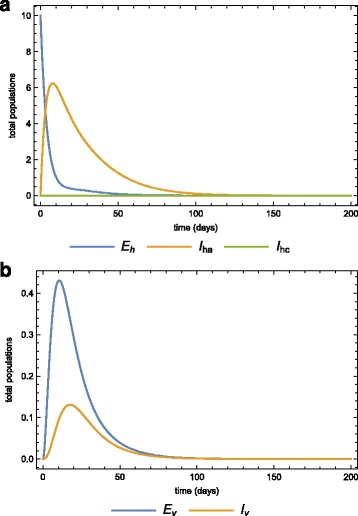
Only treatment strategy in the population. The dynamics of the **a** human, **b** vector sub-populations when there is medical treatment (prevention chemotherapy) (*φ*=0.25) and no quarantine (morbidity management and disability prevention) after the invasion of 10 infected individuals

**Fig. 8:**
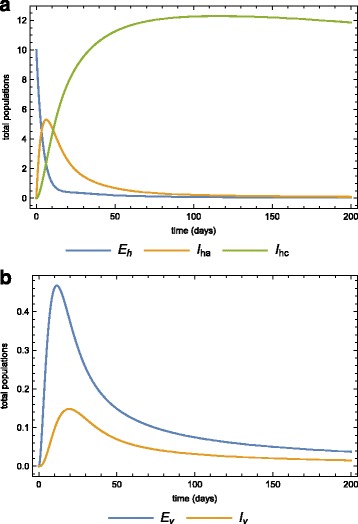
Quarantine only in the population. The dynamics of the **a** human, **b** vector sub-populations when there is quarantine (morbidity management and disability prevention) is the only strategy (*κ*=0.1) after the invasion of 10 infected individuals

Assume that a population is initially disease-free and stays at equilibrium. This population is then infiltrated by 10 infected individuals in the latent state. At this point we assume that all the mosquitoes are also disease-free. Using the parameters in Table 2, Fig. 6 a and 6 b show the dynamics of the human and vector populations when there is no treatment or quarantine (selective treatment) (*φ*=*κ*=0). The basic reproduction number is 0.482, which means that the disease will eventually die out even if there is no intervention. Fig. 7 and 7 b show the same dynamics as Fig. 5 a and 5 b when there is treatment of infected-acute individuals but no quarantine. Figure 8 a and 8 b show the effects of using quarantine of infected-chronic individuals as the only control measure. A strategy that uses both treatment and quarantine (selective treatment) is better than using only treatment or quarantine).

**Fig. 9:**
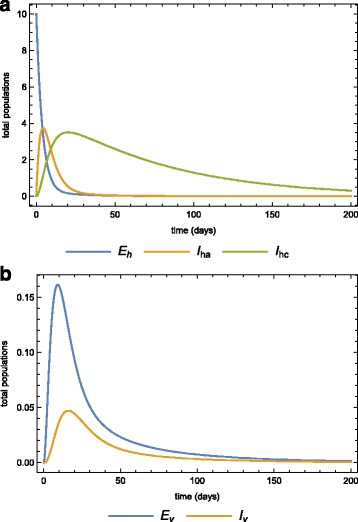
Effects of treatment. The dynamics of the **a** human, **b** vector sub-populations when there is medical treatment (prevention chemotheraphy) and the treatment reduces *𝜗* by 50*%*

The cases in Figs. 6 a, 7 and 8 b assume that the treatment given to acute-infected individuals does not affect the transmission parameters other than the screening parameter *n* and treatment parameter *φ*. The basic reproduction number depends on these two parameters and so they do affect initial disease transmission and consequently the endemic status of the disease. If patients are given medication that reduces the microfilariae in the lymphatic vessels and nodes, then the success rate of microfilariae transmission from humans to susceptible mosquitoes will decrease. Effective treatment is expected to decrease the number of microfilariae, and thus its transmission success rate from humans to susceptible mosquitoes could greatly be be altered. Assuming that individuals are further protected by some insect repellent, then the mosquito biting rate *β* could also be impacted. Consider a medication that could decrease the success rate of microfilariae transmission from humans to susceptible mosquitoes by 50*%* of its current level (*𝜗*_*v*_=0.05). Figure 9 a and 9 b show the dynamics when this effective treatment exists.

**Fig. 10:**
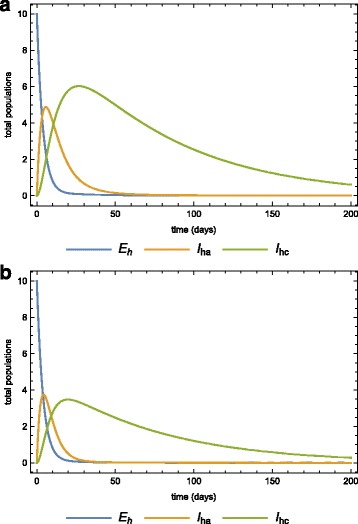
Other intervention strategies. The dynamics of the human sub-population when the **a** repellent is used in the absence of treatment, **b** repellent is used with treatment

Assume that insect repellent could decrease the insect biting rate *β* by 50*%* of its current level. Figure 10 a and 10 b show the dynamics when such a repellent is available and appropriately used. Figure 10 a shows the case when the repellent is used in the absence of medical treatment (*φ*=0) and Fig. 10 b shows the same scenario but in the presence of medical treatment (*φ*=0.3) administered at the same time as the provided insect repellent. Compared to the case when the repellent is used alone (Fig. 10 a), coupling treatment with the use of a repellent spray significantly reduces the disease outbreak (Fig. 10 b) and at the same time reduces the endemic level of the disease (changing $\mathcal {R}_{0} = 0.128$ to $\mathcal {R}_{0} = 0.094$).

Other intervention strategies such as using insecticide treated bed nets, indoor residual spraying (which both can be built into the parameters *φ* and *β*) and shortening the mosquitoes life span (increasing *μ*_*v*_) can also be considered. Consider a situation where the life span of the mosquitoes is reduced by 25*%* of the existing level with both treatment and effective repellent. The population dynamics of humans after reducing the mosquito life span is the same as the dynamics in Fig. 10 a and 10 b. However, the dynamics of the mosquito population will differ significantly for the two cases. If we decrease both values of the mosquito biting rate and the life expectancy, then there is a 50% reduction of the reproduction number (decrease from $\mathcal {R}_{0} = 0.105$ to $\mathcal {R}_{0} = 0.052$). For these parameter values, the eradication of the disease is guaranteed. In the absence of medical treatment, the basic reproduction number is slightly greater ($\mathcal {R}_{0} = 0.072$) but still less than unity. This implies that if the strategy of shortening the mosquito lifespan before a certain period of time has elapsed is applied, then the lymphatic filariasis disease is potentially bound to die out.

##### Case 2.

**Fig. 11:**
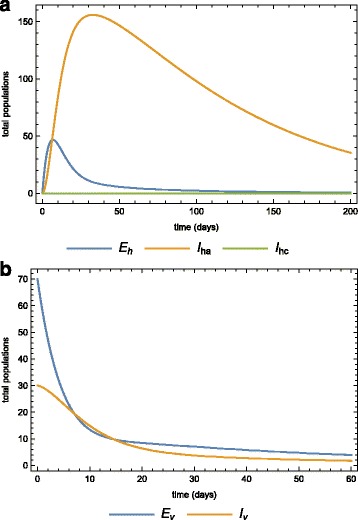
Inversion in human population by infected mosquitoes. The dynamics of the **a** human, **b** vector sub-populations when the virgin human population is invaded by infected mosquitoes

**Fig. 12:**
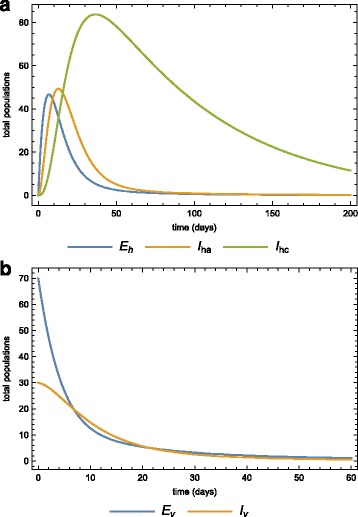
New infection in a treated and quarantined population. The dynamics of the **a** human, and vector sub-populations when the virgin human population is invaded by infected mosquitoes. In this case, there is medication (prevetion chemotheraphy) and quarantine (morbidity management and disability prevention)

In the previous section we assumed that a disease-free population is invaded by acute-infected humans. We now consider the case where a virgin population is invaded by 70 exposed and 30 infected mosquitoes from an endemic area. Figure 11 a and 11 b show the dynamics of both the human and vector populations after the invasion of the infected mosquitoes with no medication or quarantine. Employing the same intervention strategies as before, treating acute-infected and isolating some, Fig. 12 a and 12 b depict the human and mosquito populations dynamics. Any of these strategies significantly reduces the basic reproduction number.

## Discussion and conclusions

A mathematical model of the transmission dynamics of lymphatic filariasis incorporating both the human and mosquito vector was formulated and stability of equilibria and sensitivity analysis were investigated. Numerical simulations were provided to support the theoretical results. Control of infections was analyzed through two intervention strategies, namely medical treatment (prevention chemotherapy) and quarantine (selective treatment for morbidity control). The model system was globally stable and thus the phenomenon of backward bifurcation was never observed [26].

By evaluating the sensitivity indices of the reproduction numbers, we were able to identify parameters for which the model system was most sensitive. We found that the mosquito death rate was the most sensitive parameter. By also analyzing the basic reproduction number, it was shown that combined intervention strategies could lead to lymphatic filariasis elimination in the community.

The proposed model is not exhaustive and can be refined and/or extended in various ways. For instance, the emergence of drug-resistant strains of pathogens is an increasing threat to eradication of infectious diseases. Aggressive treatment might lead to drug resistance and it is worth exploring how this could affect the transmission dynamics of the disease. Also, patients’ compliance could be incorporated into the model system by assuming that only a small portion of individuals in the treatment class adhere to complete treatment, while a small proportion that do not adhere move quickly to the drug resistant class. Model extension could also address climate change since it is considered as a contributor to re-emergence of vector-borne diseases. Heavy rains and global temperature rising provide a conducive habitat for mosquitoes. Future studies could include these external factors and also consider co-infections of individuals with two types of worms.

For mathematical tractability we made several assumptions. Therefore our results are based on the formulation of the model. However, However the research undertaken enables us to gain valuable insights into lymphatic filariasis and the effectiveness of intervention strategies being implemented.
